# Complement 5a Receptor deficiency does not influence adverse cardiac remodeling after pressure-overload in mice

**DOI:** 10.1038/s41598-017-16957-3

**Published:** 2017-12-06

**Authors:** Judith J. de Haan, Lena Bosch, Anouska Borgman, Marissa Bastemeijer, Maike A. D. Brans, Sander M. van de Weg, Dominique P. V. de Kleijn, Joost P. G. Sluijter, Hamid el Azzouzi, Saskia C. A. de Jager

**Affiliations:** 1 Laboratory of Experimental Cardiology, University Medical Center Utrecht, Utrecht, The Netherlands; 2 Department of Vascular Surgery, University Medical Center Utrecht, Utrecht, The Netherlands; 3 Department of Cardiology, University Medical Center Utrecht, Utrecht, The Netherlands; 4 Netherlands Heart Institute, Utrecht, The Netherlands; 5 Utrecht University, Utrecht, The Netherlands; 6 UMC Utrecht Regenerative Medicine Center, Utrecht, The Netherlands; 7 Laboratory of Translational Immunology, Department of Immunology, University Medical Center Utrecht, Utrecht, The Netherlands

## Abstract

Hypertension is one of the most common risk factors for the development heart failure in the general population. Inflammation plays a central role in this adverse remodeling and eventually to the development of heart failure. Circulating levels of Complement factor 5a (C5a) are increased in hypertensive patients and the C5a receptor is associated with the presence of cardiac fibrosis and inflammation in an experimental hypertension model. To test if C5aR is involved in adverse cardiac remodeling following pressure-overload, we induced transverse aortic constriction (TAC) in wildtype and C5a receptor deficient mice (C5aR^−/−^). Six weeks after TAC, C5aR-/- animals showed a similar degree of cardiac hypertrophy and decrease in cardiac function as wild type mice (End Systolic Volume; 50.30±5.32 µl vs. 55.81±8.16 µl). In addition, other features of adverse cardiac remodeling like cardiomyocyte cell size (WGA staining), fibrosis (picrosirius red staining) or collagen degradation (matrix metalloproteinase activity assay) did not differ either. In conclusion, full body C5aR deficiency does not affect adverse cardiac remodeling after pressure-overload. However, our finding are in contrast with C5a inhibition studies. Our observations do present the role of C5a-C5aR in adverse cardiac remodeling and heart failure as controversial at the least.

## Introduction

Heart failure (HF) is a growing major worldwide clinical problem and it has a poor 5-year survival^1^. Next to myocardial infarction, hypertension is one of the most common risk factors for the development of HF. It has a high prevalence, 75–91% of HF cases had prior hypertension and the attributable risk for HF is accounted by hypertension in 59% in women and 39% in men in the Framingham Heart Study and Framingham Offspring Study^2,3^. In patients this chronical hypertension leads to adverse remodeling of the myocardium. Myocardial fibrosis is one of the primary pathways involved in adverse cardiac remodeling. In hypertension related cardiac remodeling this is mainly characterized by the presence of interstitial fibrosis^4^. These processes culminate in deterioration of cardiac function, which eventually leads to HF^5^.

A mostly neglected important pathway in cardiac remodeling is inflammation. Inflammation by itself can cause damage to the myocardium and as such can contribute to the progression of HF, while HF on its turn sustains the inflammatory environment in the heart causing a vicious cycle^6^. Subsequently, the inflammatory response, involving inflammatory factors and leukocytes, are thought to play an important role in the development of myocardial fibrosis^7^. Monocytes are actively recruited to the damaged myocardium, where they differentiate into macrophages and release pro-fibrotic mediators^8^. In addition, T-lymphocytes also play a pivotal role in the development of non-ischemic HF^9^ by the release of cytokines and their pro-fibrotic properties.

The complement system is part of the innate immune system and activation occurs through three different pathways, which all lead to the cleavage of complement 5(C5) to C5a and C5b^10^. C5a is a pro-inflammatory polypeptide, also referred to as an anaphylatoxin. C5a receptor (C5aR) is its most prominent receptor and is expressed on various leukocytes, such as macrophages and T-lymphocytes^11^. The other receptor for C5a, C5L2, is found on similar cell types, albeit expressed at much lower levels^12^. C5a binds with similar affinity to both receptors^11^.

C5a can exert many inflammatory functions, such as the attraction of leukocytes and stimulating the release of cytokines^11^. In addition to its expression on leukocytes, C5aR is also expressed on non-myeloid cells like fibroblasts^13,14^. In several fibrotic disease models, including liver, kidney and lung fibrosis, C5a – C5aR interaction is important for the development of fibrosis^15–18^. Furthermore, hypertensive patients have increased serum levels of C5a. Interestingly, the absence of the C5aR reduced cardiac fibrosis and inflammation in an angiotensin II (AngII)- induced hypertension mouse model^19^. Also in an ischemia-reperfusion injury model, C5aR^−/−^ mice showed reduced inflammation^20^. We therefore hypothesized that deficiency of C5aR would ameliorate pressure-overloaded adverse cardiac remodeling induced by transverse aortic constriction (TAC), accompanied by a reduced inflammatory and fibrotic response. In this study, C5aR knock-out (C5aR^−/−^) and wild type mice were subjected to TAC for 6 weeks and cardiac function was assessed by echocardiography, followed by histological analyses.

## Materials and Methods

### Animals

Male Balb/cAnCrl WT and Balb/cAnCrl C5aR^−/−^ littermates (10–12 weeks old, weight 25–30 g) from our own breeding facility (originally from Jackson laboratory) were used for the TAC model. All mice were conventionally housed in filter top cages with Aspen Woodchip bedding and with a plastic shelter with a light/dark cycle of 12/12 hours and had food and water ad libitum. A blinded researcher performed surgery on block randomly assigned animals. Technicians and observers performed the respective operations, data acquisition and analyses in a blinded fashion. Animals were monitored during the experiments by assessing weight loss and by checking physical appearance. All animal experiments were approved by the Ethical Committee on Animal Experimentation of the University Medical Center Utrecht (Utrecht, the Netherlands) and conform to the ‘Guide for the care and use of laboratory animals’.

### Transverse aortic constriction

Genotype of the animals was confirmed before the experimental procedure (see supplementary materials). Surgeries were performed in the morning and afternoon in a dedicated mouse operation room by an experienced surgeon^21^. Anaesthesia was induced by intraperitoneal (i.p.) injection of medetomidinehydrochloride (1.0 g/kg body weight), midazolam (Dormicum®, Roche, 10.0 mg/kg) and fentanyl (Janssen-Cilag, 0.1 mg/kg). These anaesthetics were preferred over cardioprotective propofol or volatile anaesthetics (e.g. isoflurane)^22^. Mice were intubated and connected to a respirator with a 1:1 oxygen-air ratio (175 strokes/minute, 250 µl stroke volume). A core body temperature of 37 °C was maintained during surgery by continuous rectal temperature monitoring and an automatic heating blanket. The aortic arch was reached between two ribs after a midline incision in the anterior neck. Transverse aortic constriction was placed between brachiocephalic artery and the left common artery against a blunt 27-gauge needle with a 7–0 silk suture followed by prompt removal of the needle. Sham operated mice underwent the same procedure without aortic constriction. Correct placement of the TAC was confirmed after 7 days by measuring the flow in the aortic arch and in the carotid arteries (flow ratio between left and right carotid ≥5 were included). For 1 week survival 4 WT and 4 C5aR^−/−^ mice endured TAC surgery and 2 WT and 2 C5aR^−/−^ were sham operated. All operated animals survived short term (1 week) follow-up, while for long term follow-up (6 weeks survival) ≈13% (2 out of 16) of WT and ≈22% (4 out of 18) of C5aR^−/−^ died as a consequence of heart failure development. All (6 WT and 6 C5aR^−/−^) of the sham operated animals survived both short term and long term follow-up. (see flow chart in supplementary material).

### Echocardiography

At baseline, 7, 14, 35 and 42 days after TAC in the morning or afternoon, anesthesia was induced by inhalation of 2.0% isoflurane in a mixture of oxygen/air (1:1). Echocardiography was performed to assess cardiac geometry and function. Heart rate, respiration and body temperature were constantly monitored and body temperature was kept between 36.0 and 38.0 °C using heating lamps. Two-dimensional images were recorded on the short axis of the heart on multiple levels in both end systole and end diastole and with respiratory triggering. These images were subsequently used for complete 3D reconstruction of the heart. Image acquisition and analyses were performed using the dedicated Vevo® 2100 System and Software (Fujifilm VisualSonics Inc., Toronto, Canada). For global longitudinal strain (GLS) analysis on long axis images TomTec 2D cardiac performance Analysis (TomTec GMBH, Unterschleissheim, Germany) was used.

### Termination

At termination, body weight was assessed. Mice were euthanized using sodium pentobarbital (60.0 g/kg). Blood was collected through orbital puncture in EDTA coated tubes for flow cytometry and plasma collection. The vascular system was flushed with 5 mL phosphate-buffered saline (PBS) through right ventricular puncture. Heart weight and tibia length was assessed. One half of the heart in four chamber view was formalin-fixed for 24 hours and afterwards embedded in paraffin. The other half was snap-frozen in liquid nitrogen for RNA or protein isolation and stored at −80 °C. The spleen and the mediastinal lymph nodes posterior to the heart were excised for flow cytometric analyses.

### Histology

Paraffin embedded hearts were cut in 5 µm thick sections and fixed on slides (Ultra Plus®, Thermo Scientific). Sections were dried for at least one hour at 55 °C. Before staining, sections were deparaffinized and rehydrated (2 × 8 minutes in Ultraclear (1466, Sakura), 2 × 5 minutes in 100% EtOH (4099.9005, Klinipath), 2 × 5 minutes in 96% EtOH (Klinipath), 5 minutes in 70% EtOH (Klinipath), 5 minutes in Demi H2O). A Hematoxylin/Eosin staining was used to visualize global morphology. Quantification of collagen density was performed using picrosirius red staining. Collagen density analysis was done with circularly polarized light, converted into grey scale images and given as a percentage of the total area.

Antigen retrieval was performed by 20 minutes boiling in 10 mM citrate buffer pH 6.0 (6132-04-3, Sigma Aldrich). To examine cell size, a Wheat germ agglutinin (WGA) staining was performed. Slides were stained for 30 min with WGA-FITC labeled antibody (1:40, L4895, Sigma Aldrich) at room temperature (RT). Staining with Hoechst (1:10.000, 33342(H1399), Thermo Scientific) for 5 min was done to visualize the nuclei and Fluoromount (0100-01, SouthernBiotech) was used to cover the slides. T-cells were stained using a polyclonal rabbit-anti-human CD3 antibody (1:100, Dako, A0452) and anti-rabbit-AP Powervision (pure, DPVR-110AP, Immunologic) was used as secondary antibody. Macrophages were stained using a rat-anti-mouse MAC3 antibody (1:30, BD Pharmingen, 553322, 0.5 mg/ml). Rabbit-anti-rat-biotin (1:200, DAKO E0468, 0.84 g/L) was used as a secondary antibody and Streptavidin-AP (1:500, SA-5100) as a tertiary antibody. Liquid permanent red was used as an enzyme substrate.

To assess the amount of myofibroblasts and smooth muscle cells, αSMA-FITC (1:400, clone 1A4, F3777, Sigma) staining was performed. Incubation with the labeled primary antibody was done for 60 min at RT. Staining with Hoechst (1:10.000, 33342(H1399), Thermo Scientific) for 15 sec was done to visualize the nuclei and Fluoromount (0100-01, SouthernBiotech) was used to cover the slides.

Images of tissue sections were captured with a BX53 microscope (Olympos) and analysis was performed in a blinded fashion using CellSens (Olympus Corporation, Tokyo, Japan) or ImageJ. The latter was used to determine cell size by use of the WGA staining, with an in house developed macro tool for ImageJ. Researchers were blinded during the analysis.

### RNA isolation and cDNA synthesis

To extract RNA from heart tissue the Nucleospin RNA II-kit (740955.250, Bioke) was used according to manufacturer’s protocol. RNA concentration was measured on the Xpose^TM^ and the RNA was stored at −80 °C. Before cDNA synthesis, genomic DNA was removed by DNAse treatment with the TURBO DNA-free kit (AM1907, Ambion). cDNA was synthesized using the qScript cDNA synthesis kit (95047-025, Quanta biosciences), with 500 ng RNA per reaction. cDNA was diluted 20 times and stored at −20 °C until further use.

### Quantitive polymerase chain reaction (qPCR)

The expression levels of pro-collagen 1(Pro-Col1), C5L2 were analysed by qPCR. qPCR was performed in duplo with SybrGreen (1708880, Bio-Rad) at a Bio-Rad CFX96 Real Time system. A heat start of 95 °C for 3 minutes, 40 cycli of 95 °C for 10 seconds, 50–65 °C for 30 seconds and 72 °C for 30 seconds was the protocol that was used. A melt curve was included to confirm specificity. The mRNA expression was determined relative to the average expression of two household genes P0 and RPL27. Primer sequences are depicted in Table 1.

**Table 1:** primer sequences of housekeeping genes and genes of interest.

Gene	Sequence forward	Sequence reverse
P0	5′-GGACCCGAGAAGACCTCCTT-′3	5′-GCACATCACTCAGAATTTCAATG-′3
RPL27	5′-CGCCCTCCTTTCCTTTCTGC-′3	5′-GGTGCCATCGTCAATGTTCTTC-′3
α-SMA	5′-GCTATGCCTTGCCCATGC-′3	5′-TTCCGATGGTGATCACTTGCC-′3
C5aR	5′-GCTCAAAGTGGTGATGGC-′3	5′-CACAGCAGTTGATGTAGGC-′3
C5L2	5′-ACCACCAGCGAGTATTATGACT-′3	5′-GCTGCATACAGCACAAGCA-′3
Col1	5′-TCAAGGTCTACTGCAACATGG-′3	5′-AATCCATCGGTCATGCTCTCT-′3
Col3a1	5′-CTGGTTCTTCTGGACATCC-′3	5′-TCTTCCTGACTCTCCATCC-′3
TGF-β1	5′-ACTACTATGCTAAAGAGG-′3	5′-TTGTTGCTATATTTCTGG-′3

### Flow cytometry

To get a single cell suspension, lymph nodes and spleens were put through a 40 µm filter. White blood cell counts of blood were measured on a Cell-Dyn 3200 automatic cell counter (Abbott Diagonstics).

Fifty µL of blood, spleen or lymph node cell suspension was added to 100 µL of antibody mixture containing different cell surface markers (supplemental table I) and incubated for 30 min in the dark at RT. After red blood cell lysis using Optilyse C (Beckman Coulter)10 minutes at RT, the samples were measured on the flow cytometer (Gallios, Beckman Coulter, Marseille, France). Kaluza Analysis Software 1.2 was used to analyze the data. Percentage of total cell number of neutrophils, monocytes and T cells as assessed by flow cytometry are presented.

### Zymogram

Matrix Metalloproteinase (MMP) activity in the myocardium was examined by gelatin zymography according to manufacturer's protocol. Myocardial samples were homogenized in Roche lysis buffer and protein concentration was measured using BCA Protein Assay Kit (Thermo Scientific). Twenty µg of myocardial extract diluted in 4x Laemli buffer (25 M Tris-HCl, 8% SDS, 40% Glycerol, 0.004% Broom phenol Blue, pH 6.8) was loaded on precast gelatin gels (EC6175BOX, Invitrogen). After running, the gels were washed in 2.5% Triton X-100 followed by overnight incubation at 37 °C in Brij-solution (50 mM Tris-HCl pH 7.4; 10 mM CaCl2 (Merck, Whitehouse Station, USA); 0.05% Brij35 (Sigma-Aldrich)). After incubation, the gels were fixed and stained with Coomassie blue (0.1% Coomassie Brilliant blue R-250 (Bio-Rad), 25% methanol and 15% acetic acid (both from Sigma-Aldrich)) and subsequently destained (in 25% methanol and 15% acetic acid) until clear bands appeared against blue background. Pictures were taken on a Chemidoc XRS + imager system. Images were analyzed using ImageLab (Bio-Rad) software.

### Statistical analysis

For group size calculation we defined end systolic volume (ESV) (µl) as primary outcome, representing a surrogate marker of HF. We calculated our primary outcome on the diameter of the left ventricle, however we represented volumes of the left ventricle in this manuscript. We analyzed our data using 3D reconstruction, what was not yet available at the time of designing the experiment, nevertheless this is more precise and will lead to an even higher power than we initially planned. For calculating the sample size, we took a power of 90%, an alpha of 0.05 and a standard deviation of 0.29 mm^23^. Our desired effect was 0.40 mm. Corrected for multiple testing (four groups in total: two experimental groups; WT TAC, and C5aR^−/−^ TAC, and two control groups; WT Sham and C5aR^−/−^ Sham) this resulted in a minimum of n = 12 per group for 6 weeks follow-up, with a single animal as experimental unit. Due to anticipated mortality we operated in total N = 16 WT and N = 18 C5aR^−/−^ TAC mice. In the WT TAC group, 2 mice died and one was excluded based on carotid flow ratios. In the C5aR^−/−^ TAC group, 4 mice died and 1 was excluded based on carotid flow ratios. For 1 week follow-up, we included 4 WT and C5aR^−/−^ TAC and 2 WT and C5aR^−/−^ Sham. Our secondary outcomes are end diastolic function (EDV) (µl), global longitudinal strain (GLS) (%), histological stainings (count or %), zymograms (relative band intensity) and qPCRs (ΔΔCq).Because of technical failure, some samples were excluded for analysis of secondary outcome measures. Data are presented as mean ± standard deviation (Sd). Student T-test was used for EDV and ESV, heart weight/tibia length, WGA, picrosirius red staining, α-SMA and qPCRs. Statistical analyses were performed using GraphPad Prism 6 and p ≤ 0.05 was considered statistically significant.

## Results

### Survival, cardiac hypertrophy, and cardiac function are comparable between WT and C5aR^−/−^ mice following TAC

**Figure 1:**
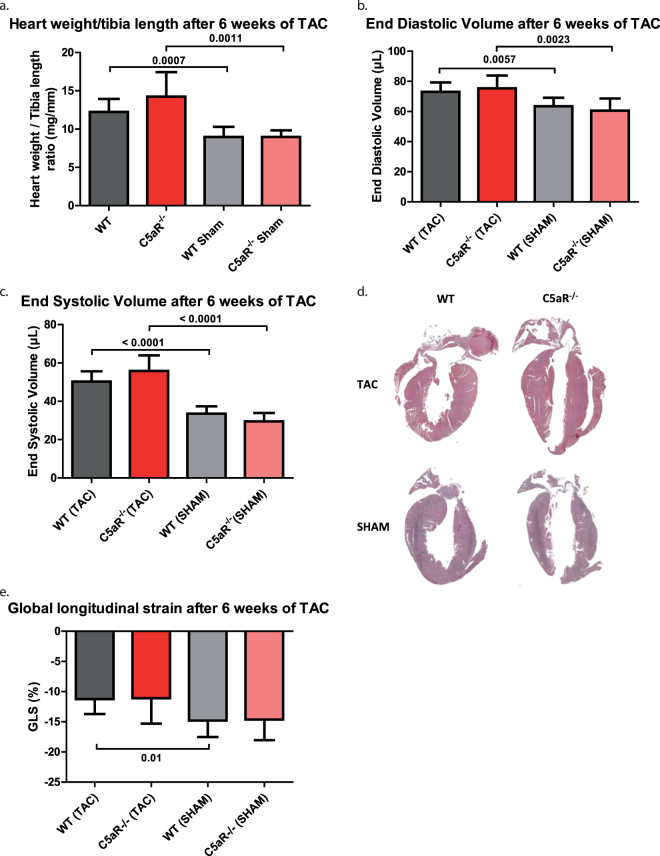
Adverse cardiac remodeling is not altered in C5aR^−/−^ mice after 6 weeks of TAC. There is no difference in cardiac hypertrophy between WT and C5aR^−/−^ (**a**) or EDV (**b**) and ESV (**c**). Global cardiac morphology does not appear to be altered based on HE staining (**d**). GLS is decreased after TAC but not different between WT and C5aR^−/−^ (**e**). N = 13 animal for WT and C5aR^−/−^ TAC and N = 6 animals for WT and C5aR^−/−^ Sham,. *WT: Wild-type; C5aR* ^−/−^ *:C5aR deficient*.

Within 42 days after TAC, cardiac death occurred in ≈13% (2 out of 16) of WT mice and ≈22% (4 out of 18) of C5aR^−/−^ mice. One WT and 1 C5aR^−/−^ TAC operated mouse were excluded based on carotid flow ratio <5. The hearts of both WT and C5aR^−/−^ mice that underwent TAC surgery displayed cardiac hypertrophic remodeling, as shown by an increase in heart weight/tibia length (WT: 8.98 ± 1.31 vs. 12.24 ± 1.70 mg/mm, p = 0.0007; C5aR^−/−^: 8.98 ± 0.86 vs. 14.25 ± 3.19 mg/mm, p = 0.0011, Fig. 1a)) compared to sham operated counter groups. No significant difference was found between the TAC operated WT and C5aR^−/−^ mice. Similarly, EDV and ESV was increased at 42 days after TAC in both WT and C5aR^−/−^ mice compared to sham operated mice (EDV; WT: 63.49 ± 5.67 vs. 73.03 ± 6.30 µl, p = 0.0057; C5aR^−/−^: 60.58 ± 8.05 vs. 75.37 ± 8.52 µl, p = 0.0023, Fig. 1b,c) (ESV; WT: 33.56 ± 3.83 vs. 50.30 ± 5.32 µl, p < 0.0001; C5aR^−/−^: 29.49 ± 4.40 vs. 55.81 ± 8.16 µl, p < 0.0001< Fig. 1b). Moreover, both cardiac volumes did not differ between WT and C5aR^−/−^ mice that underwent TAC. GLS also worsened after 42 days of TAC (GLS; WT: -14.79±2.73 vs. −11.23 ± 2.49 %, p = 0.0136; C5aR^−/−^: −14.64 ± 3.41 vs. −11.09 ± 4.2, p = 0.0938), but did not differ between the genotypes. Overall morphology showed no apparent differences between WT and C5aR^−/−^ mice as indicated by H&E staining (Fig. 1d).

### Leukocyte levels in the heart, blood, spleen and lymph nodes are not altered after TAC in the absence of C5aR

**Figure 2:**
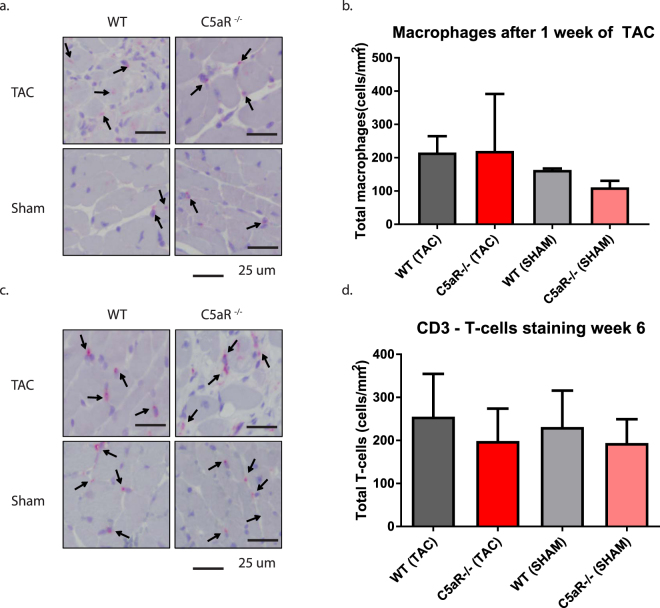
Influx of leukocytes in the heart after TAC is not altered in C5aR^−/−^ mice. Macrophage influx was assessed with MAC-3 staining (**a**) and quantified as cells/mm^2^ (**b**) N = 3 for WT TAC, N = 4 for C5aR^−/−^ TAC and N = 2 for WT and C5aR^−/−^ Sham. T-cell influx was assessed with CD3 staining (**c**) and quantified as cells/mm^2^(**d**). N = 11 for WT and C5aR^−/−^ TAC and N = 6 for WT and C5aR^−/−^ Sham.

The C5aR has been shown to influence the inflammatory response in various cardiac and non-cardiac diseases^19,20,24^. Therefore we performed flow cytometric analysis on blood, spleen and (draining) lymph nodes at 7 days and 42 days after TAC. Total white blood cell count did not differ between WT and C5aR^−/−^ mice (data not shown), therefore we depicted the leukocyte subpopulations as percentages of total cell count. In addition, we did not observe any difference in total leukocyte counts or subset distribution between WT and C5aR^−/−^ mice (Tables 2–4). We also studied the number of macrophages in the heart after TAC using immunohistochemistry. Macrophage influx was slightly, though not significantly, increased after 1 week of TAC (WT: 163 ± 5 vs. 216 ± 49 cells/mm^2^, p = 0.250; KO: 111 ± 20 vs. 220 ± 171 cells/mm^2^, p = 0.44, Fig. 2a,b). The amount of T-cells was not different after 6 weeks of TAC in WT or C5aR^−/−^ mice (WT: 231 ± 85 vs. 255 ± 99 cells/mm^2^, p = 0.625; KO: 194 ± 56 vs. 199 ± 75 cells/mm^2^, p = 0.892, Fig. 2c,d).

**Table 2:** blood leukocytes.

Cell subtype (%)	Sham				TAC			
	1 week		6 weeks		1 week		6 weeks	
	WT (N = 2)	C5aR^−/−^ (N = 2)	WT (N = 2)	C5aR^−/−^ (N = 3)	WT (N = 4)	C5aR^−/−^ (N = 4)	WT (N = 12)	C5aR^−/−^(N = 8)
Neutrophils	21.2 ± 4.6	37.8 ± 27.	16.5 ± 3.8	5.5 ± 3.6	23.5 ± 6.4	44.6 ± 20.2	12.2 ± 4.0	11.4 ± 6.9
Ly6C High monocytes	0.7 ± 0.2	0.9 ± 0.3	1.2 ± 0.3	0.6 ± 0.2	1.4 ± 0.6	1.1 ± 0.4	1.7 ± 0.9	1.6 ± 0.8
Ly6C Low monocytes	1.4 ± 0.1	1.2 ± 0.1	2.0 ± 0.6	2.1 ± 0.1	1.8 ± 0.7	1.7 ± 0.3	3.4 ± 0.9	3.1 ± 0.7
Dendritic Cells	1.1 ± 0.4	0.9 ± 0.2	1.2 ± 0.2	1.3 ± 0.2	1.7 ± 1.0	1.2 ± 0.3	1.8 ± 0.7	1.6 ± 0.7
CD4 + T Cells	22.1 ± 3.4	21.9 ± 3.3	27.9 ± 4.1	34.8 ± 2.5	21.1 ± 8.7	20.8 ± 2.7	21.8 ± 11.4	19.8 ± 10.6
Treg	4.1 ± 0.9	4.1 ± 1.2	2.2 ± 0.2	3.2 ± 0.7	8.6 ± 9.0	3.4 ± 1.2	7.3 ± 8.3	9.2 ± 8.5
CD8 + T Cells	8.7 ± 0.3	6.3 ± 3.2	13.1 ± 3.4	16.0 ± 2.5	9.0 ± 2.3	6.5 ± 1.4	12.2 ± 2.1	14.9 ± 1.3

**Table 3:** spleen lymphocytes.

Cell subtype (%)	**Sham**				**TAC**			
	1 week		6 weeks		1 week		6 weeks	
	WT(N = 2)	C5aR^−/−^ (N = 2)	WT(N = 2)	C5aR^−/−^(N = 3)	WT(N = 4)	C5aR^−/−^(N = 4)	WT(N = 12)	C5aR^−/−^(N = 8)
CD4	22.9 ± 2.2	22.1 ± 4.2	20.2 ± 1.2	23.6 ± 2.1	20.2 ± 1.2	22.8 ± 2.3	19.9 ± 2.2	20.5 ± 1.8
Treg	0.7 ± 0.1	1.0 ± 0.3	0.9 ± 0.3	0.6 ± 0.4	1.0 ± 0.1	0.8 ± 0.1	0.9 ± 0.4	1.3 ± 1.2
CD8	10.1 ± 0.1	8.3 ± 2.4	9.1 ± 1.1	10.4 ± 1.3	8.5 ± 0.8	9.9 ± 2.0	9.0 ± 1.3	9.1 ± 0.9

**Table 4:** lymph node lymphocytes.

Cell subtype (%)	Sham				TAC			
	1 week		6 weeks		1 week		6 weeks	
	WT(N = 2)	C5aR^−/−^(N = 2)	WT(N = 1)	C5aR^−/−^(N = 3)	WT(N = 4)	C5aR^−/−^(N = 4)	WT(N = 12)	C5aR^−/−^(N = 8)
CD4	13.9 ± 1.5	16.4 ± 12.6	21.9	27.5 ± 5.2	23.5 ± 7.8	33.2 ± 2.3	21.7 ± 7.3	28.6 ± 5.8
Treg	0.7 ± 0.1	0.6 ± 0.05	0.9	0.7 ± 0.2	0.7 ± 0.2	0.8 ± 0.3	1.0 ± 0.3	1.0 ± 0.5
CD8	7.7 ± 0.9	6.3 ± 4.3	9.4	11.9 ± 3.0	13.1 ± 5.1	15.2 ± 1.0	9.5 ± 3.4	12.3 ± 3.7

### Fibrosis and MMP activity is increased after TAC, but similar between WT and C5aR^−/−^ mice

**Figure 3:**
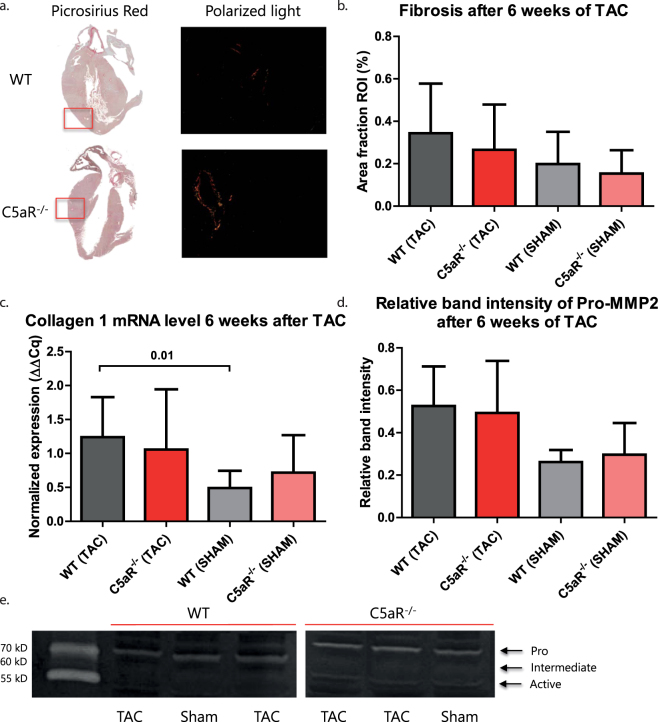
Matrix remodeling is increased upon TAC. Collagen deposition was assessed with picrosirius red staining (**a**) and depicted as percentage of myocardial area (**b**) N = 11 animals for WT and C5aR^−/−^ TAC, N = 6 for WT C5aR^−/−^ Sham. mRNA levels of collagen I are increased upon TAC but not different between WT and C5aR^−/−^ mice (**c**). N = 11 animals for WT and C5aR^−/−^ TAC, N = 5 for WT C5aR^−/−^ Sham. Quantification of zymography gels of MMP2 activity is shown (**d**) and two cropped gels are displayed (**e**). N = 9 animals for WT TAC, N = 11 for C5aR^−/−^ TAC, N = 2 for WT Sham and N = 5 for C5aR^−/−^ Sham. *WT: Wild-type; C5aR* ^−/−^ *:C5aR deficient*.

In general, inhibition of C5aR caused a decrease in fibrotic events in several fibrosis models^15,16^. Concomitant with these findings, deficiency of C5aR and the use of an C5aR antagonist in an AngII hypertension model lead to a decrease in fibrosis in the heart^19,25^. Therefore, we assessed the level of cardiac fibrosis after 6 weeks of TAC. Collagen deposition was slightly increased at 42 days after TAC compared to sham (WT: 0.198 ± 0.152 vs. 0.343 ± 0.234, p = 0.157; KO: 0.1533 ± 0.110 vs. 0.266 ± 0.214, p = 0.241, Fig. 3a,b), although it did not reach statistical significance (Fig. 2b). To study if matrix remodeling was affected by the absence of C5aR, we also looked at the mRNA levels of collagen and the activity of Matrix Metalloproteinases (MMPs). MMPs are involved in matrix homeostasis and are particularly important for collagen degradation. mRNA levels of collagen I were significantly increased upon TAC in WT mice (0.491 ± 0.253 in WT sham vs. 1.241 ± 0.589 in WT TAC, p = 0.0174, Fig. 2c), but not in C5aR^−/−^ deficient mice (0.718 ± 0.552 in C5aR^−/−^ sham vs 1.057 ± 0.888 C5aR^−/−^ TAC, p = 0.447, Fig. 3c). No differences were found between WT and C5aR^−/−^ mice for collagen deposition and Col-I mRNA levels (Fig. 3b,c). To assess if the absence of C5aR affected MMP regulation, zymography was performed to measure MMP activity. Intermediate and active MMP2 were found to be below detection level. There was a mild, albeit non-significant, increase in pro-MMP2 after 6 weeks of TAC in both WT (0.262 ± 0.057 vs. 0.525 ± 0.187, p = 0.0889) and C5aR^−/−^ mice (0.297 ± 0.149 vs. 0.493 ± 0.246, p = 0.124), however pro-MMP2 levels were comparable between WT and C5aR^−/−^ mice after TAC (Fig. 3d,e).

### Cardiomyocyte hypertrophy does not differ between WT and C5aR^−/−^ mice

**Figure 4:**
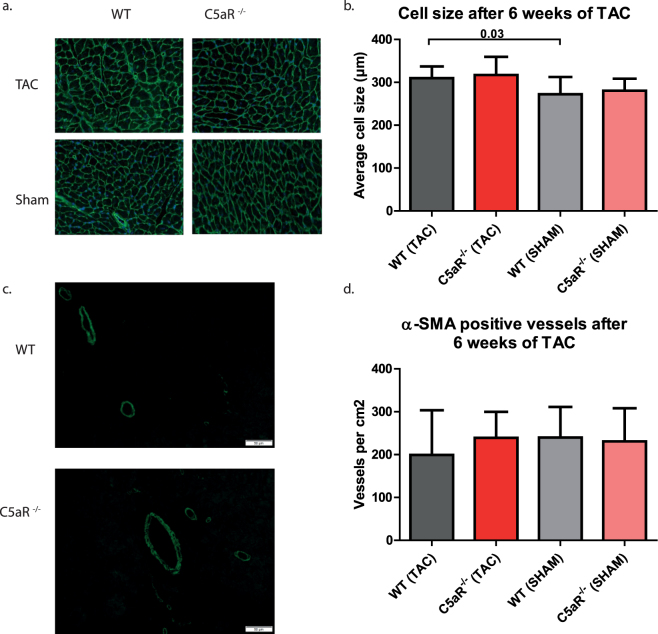
The level of hypertrophy of the cardiomyocytes and density of αSMA positive vessels is not different between WT and C5aR^−/−^ mice. WGA staining was performed to assess cardiomyocyte cell size (**a**). Cell size increased upon 6 weeks of TAC, but was similar in WT and C5aR^−/−^ mice N = 11 animals for WT TAC, N = 10 for C5aR^−/−^ TAC, N = 6 for WT and C5aR^−/−^ Sham (**b**). αSMA positive vessels (**c**) are not altered in density after TAC or between WT and C5aR^−/−^ mice (**d**).N = 11 for WT and C5aR^−/−^ TAC, N = 6 for WT and C5aR^−/−^ Sham. *WT: Wild-type; C5aR* ^−/−^ *:C5aR deficient*.

Pressure-overload on the heart induces cardiomyocyte hypertrophy. To study whether the absence of C5aR affected this, a WGA staining was performed. WGA demarcates the cell membrane of individual cells (Fig. 4a) and it showed a significant increase in cell size in WT mice (272 ± 40 µm^2^ in sham operated mice vs. 310 ± 27 in TAC mice, p = 0.0355, Fig. 4b) and a trend towards an increase in C5aR^−/−^ mice (280 ± 28 µm^2^ in sham operated mice vs. 317 ± 43 µm^2^ in TAC mice, p = 0.0828, Fig. 4b) upon 6 weeks of TAC. However, no difference was found between TAC operated WT and C5aR^−/−^ mice (p = 0.648, Fig. 4b).

### The density of α-SMA positive vessels is not altered after TAC

Alterations in vessel formation have been shown to be present after TAC^23^ and the complement system has been described to be involved in angiogenesis^26^. For this, we stained α-SMA to quantify the amount of blood vessels in the heart (Fig. 4c). We observed no difference in the amount of α-SMA positive vessels at 42 days after TAC in WT (WT: 240 ± 71 vessels/cm^2^ in sham operated WT vs. 199.2 ± 104 vessels/cm^2^ in WT TAC animals, p = 0.409) compared to sham operated or C5aR^−/−^ mice (231 ± 77.6 vessels/cm^2^ in sham operated C5aR^−/−^ vs. 239 ± 61 vessels/cm^2^ in C5aR^−/−^ TAC animals, p = 0.810, Fig. 4d).

## Discussion

C5aR^−/−^ and wild type mice were subjected to TAC to induce pressure overload initiated cardiac remodeling. After 42 days of aortic constriction, we did not observe differences in adverse cardiac remodeling as seen in similar extent of cardiac hypertrophy, loss of cardiac function or biochemical parameters in the C5aR^−/−^ and wild type mice.

C5a has been shown to be involved in inflammation and fibrosis through activating fibroblasts, thereby stimulating the production of collagens^16^ or increasing their migratory capacity^18^. In addition, C5a is important for the active recruitment of inflammatory cells, induction of a respiratory burst, and the release of cytokines and chemokines^11,27^. Our data are in remarkable contrast with the study of Iyer *et al*. using a C5aR antagonist in DOCA-salt hypertensive rats showing attenuation of adverse cardiac remodeling^28^. These different observations might be explained by the different animal models that were used. The DOCA-salt hypertensive rat causes cardiac hypertrophy mainly by elevating the blood pressure^29^ while the TAC model administers direct pressure-overload to the heart and causes thereby cardiac remodeling. Another explanation for the observed discrepancies might be that in our study, we used full body C5aR knock-out mice, while Iyer *et al*. used a selective C5aR antagonist and for a certain time span. A disadvantage of an antagonist approach is that the level of inhibition barely ever reaches 100% and that there can be unwanted side effects by a-specific binding. A full body knock-out approach also has limitations, for example the absence of the C5a receptor during whole life span can influence embryonic development and this could potentially influence the degree of cardiac remodeling upon TAC at a later time point. Furthermore, the receptor is absent in all cell types and unknown functions of the receptor might influence outcome. In addition, it may very well be that in our C5aR^−/−^ animals compensation mechanisms occur, such as upregulation of the C5L2 receptor, that may have not occurred in the DOCA-salt rats. To address this we determined C5L2 receptor expression in the myocardium of WT and C5aR^−/−^ animals, however mRNA expression levels determined by qPCR were undetectably low in the hearts of our mice after TAC (data not shown), and as such may not be responsible for a compensatory mechanism.

In an ischemia/reperfusion model using bone marrow chimeras, mainly the expression of C5aR on leukocytes affected the inflammatory response and the extend of cardiac damage^20^. In our model, we did not make a distinction between the expression of C5aR on circulating cells or on non-myeloid cells. It may be that the role of C5aR in leukocytes is different comparing to non-myeloid cells, which could be the reason that we do not observe altered cardiac remodeling. In addition, the usage of a C5aR antagonist may have specifically targeted circulating cells, simply as a consequence of accessibility, thereby attenuating cardiac remodeling.

As expected in the TAC model, fibrosis increased after TAC in both WT and C5aR^−/−^ mice but did not differ between WT and C5aR^−/−^ mice. A study of Zhang *et al*., however, showed a marked reduction of collagen deposition in the myocardium of C5aR^−/−^ after one week of Angiotensin-II infusion^19^, which may suggest that measuring collagen deposition after 6 weeks of TAC might be too late. However, it must be noted that one week after TAC, we also did not observe differences in picrosirius red staining between WT and C5aR^−/−^, albeit that collagen content was quite low at this time point (data not shown). Another reason may be found in the different triggers for fibrosis formation in both models. AngII can directly stimulate the production of collagen from cardiac fibroblasts^30^, and as such the effect of C5aR might be more pronounced in the AngII model, while not evident in our TAC model.

Also in other fibrotic disease models, such as lung fibrosis, specific C5aR blockade by siRNA resulted in collagen reduction^16^. In addition, C5aR antagonists also have a positive effect on collagen formation in a fibrotic kidney disease model and an AngII hypertension model^15,25^. The fact that we do not observe changes in collagen deposition, may again be related to the use of C5aR knock-out mice.

In conclusion, in our study C5aR seems not to be involved in adverse remodeling after pressure-overload (TAC model) in mice. As such, the role of C5aR in adverse cardiac remodeling remains controversial and more studies are necessary to completely uncover the exact role of the C5a axis in this process.

## Electronic supplementary material

Supplementary material
