# Supplementary material for: Complement 5a Receptor deficiency does not influence adverse cardiac remodeling after pressure-overload in mice
Source: Sci Rep. 2017 Dec 6;7:17045. doi: 10.1038/s41598-017-16957-3 (PMC5719022; doi:10.1038/s41598-017-16957-3)

Complement 5a Receptor deficiency does not influence adverse cardiac remodeling after pressure-overload in mice

Judith J. de Haan[1], MSc□; Lena Bosch[1], MD□; Anouska Borgman[1],BSc; Marissa Bastemeijer[1], MSc; Maike A.D. Brans[1,] BSc; Sander M. van de Weg[1], BSc; PhD; Dominique P.V. de Kleijn[2,3,4,],Joost P.G. Sluijter, PhD[1,5,6]; PhD; Hamid el Azzouzi[1], PhD; Saskia C.A. de Jager[1,7], PhD*

[1] Department of Cardiology, Laboratory of Experimental Cardiology, University Medical Center Utrecht, The Netherlands; [2] Department of Vascular Surgery, University Medical Center Utrecht, Utrecht, the Netherlands; [3] Department of Cardiology, University Medical Center Utrecht, The Netherlands; [4] Netherlands Heart Institute, Utrecht, the Netherlands; [5] University Utrecht, [6] UMC Utrecht Regenerative Medicine Center, Utrecht, [7] Laboratory of Translational Immunology, Department of Immunology, University Medical Center Utrecht, Utrecht, The Netherlands

□ both authors contributed equally to this work

Supplementary material

Genotyping

All mice were genotyped before the experiments. DNA was isolated from an ear tag, using a DNA extraction kit containing extraction buffer (E7526, Sigma-Aldrich), tissue preparation solution (T3073, Sigma-Aldrich) and a neutralizing buffer (N3910, Sigma-Aldrich). A PCR was performed according to this protocol: 94°C for 30 seconds, 35 cycli of 30 seconds at 94°C, 1 minute at 63°C and 1 minute 72°C ending with 2 minutes at 72°C. The following primers were used: 5'-GGTCTCTCCCCAGCATCATA-'3 (wild type forward), 5'-GGCAACGTAGCCAAGAAAAA-'3 (common primer) and 5'-GCCAGAGGCCACTTGTGTAG-'3 (mutant forward). The PCR products were run at a 2% agarose gel and visualized with a ChemiDoc XRS+ and analyzed with ImageLab software. A band at 386 kb are expected for wild type mice, a band at 244 kb for C5aR$^{-/-}$ mice and a double band at 386 kb and 244 kb for heterozygote mice.

Supplementary Figure 1: Example of genotyping

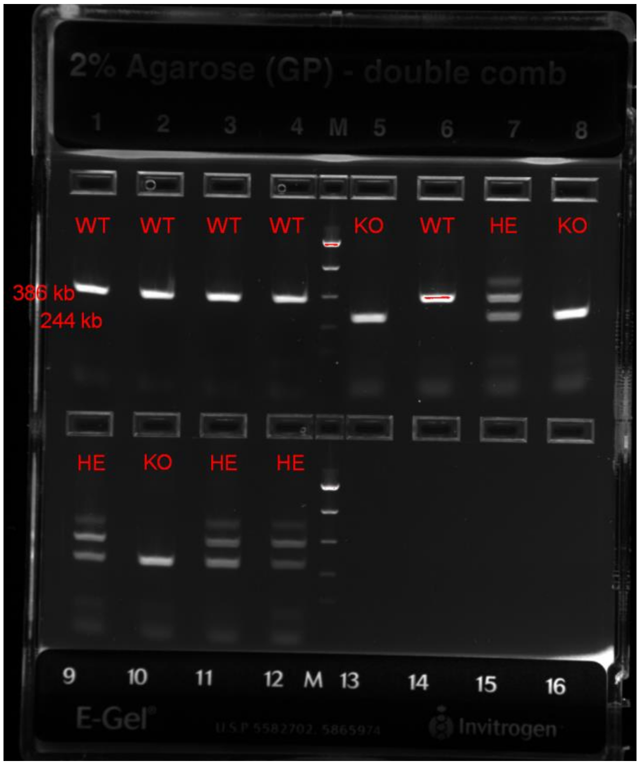

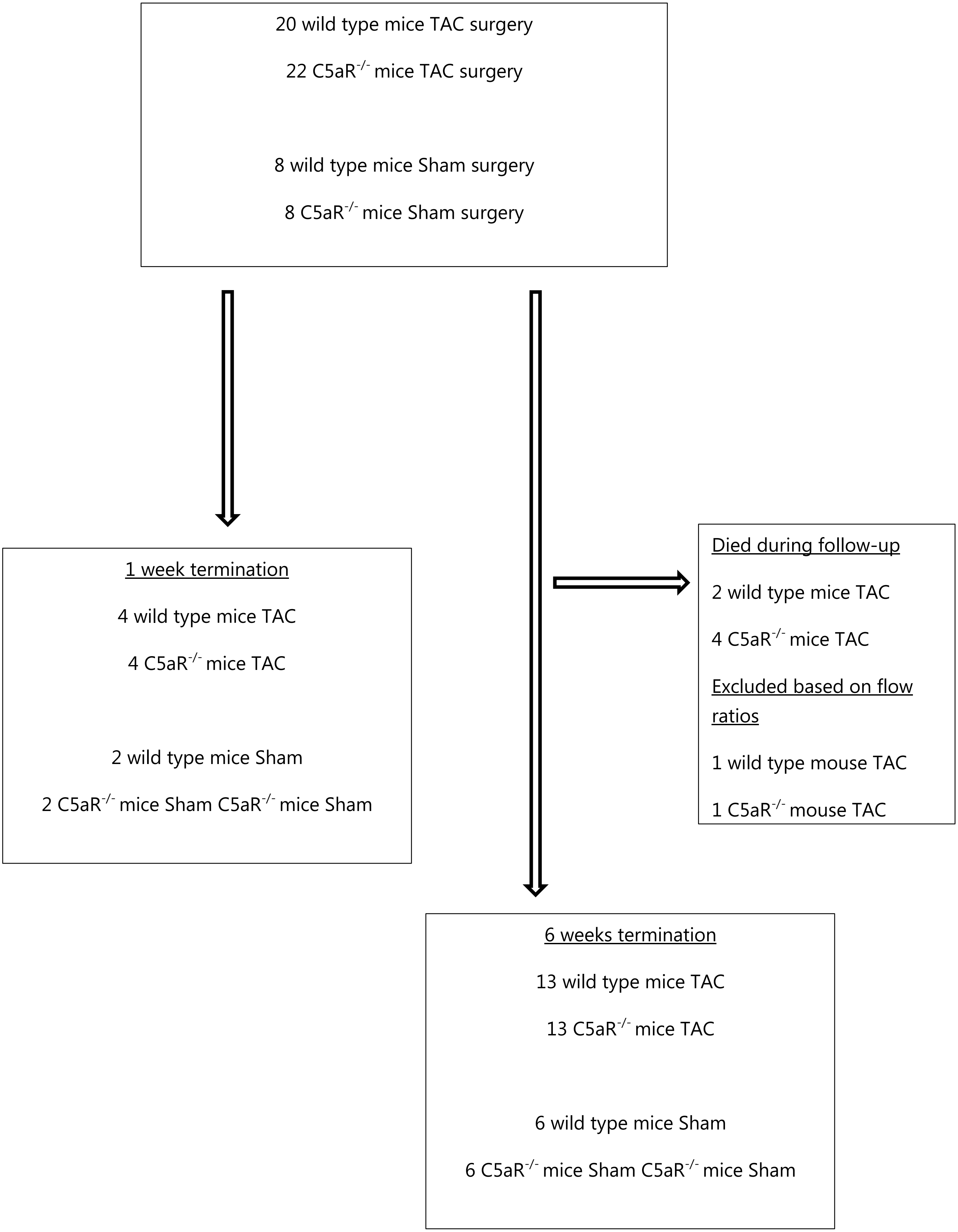
20 wild type mice TAC surgery
22 C5aR-/- mice TAC surgery
8 wild type mice Sham surgery
8 C5aR-/- mice Sham surgery
1 week termination
4 wild type mice TAC
4 C5aR-/- mice TAC
2 wild type mice Sham
2 C5aR-/- mice Sham C5aR-/- mice Sham
Died during follow-up
2 wild type mice TAC
4 C5aR-/- mice TAC
Excluded based on flow ratios
1 wild type mouse TAC
1 C5aR-/- mouse TAC
6 weeks termination
13 wild type mice TAC
13 C5aR-/- mice TAC
6 wild type mice Sham
6 C5aR-/- mice Sham C5aR-/- mice Sham

Supplementary figure 2. Number of mast cells in the heart after 6 weeks of TAC

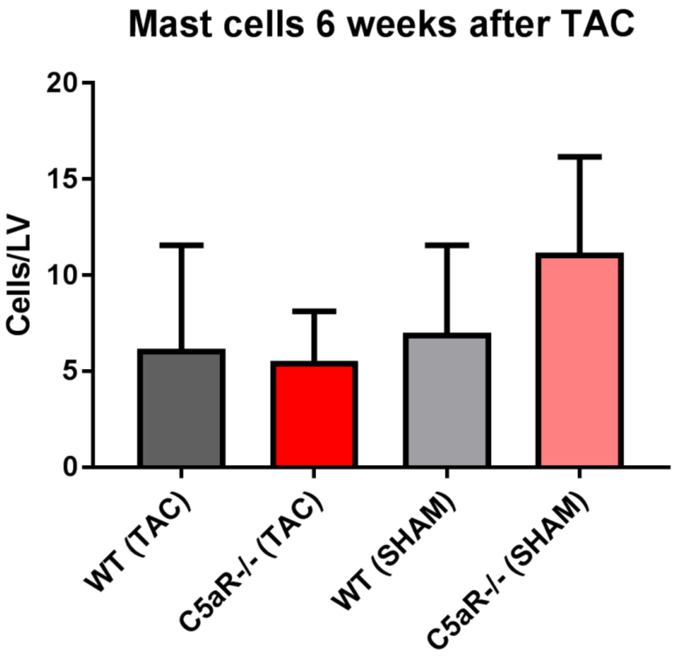

WT TAC

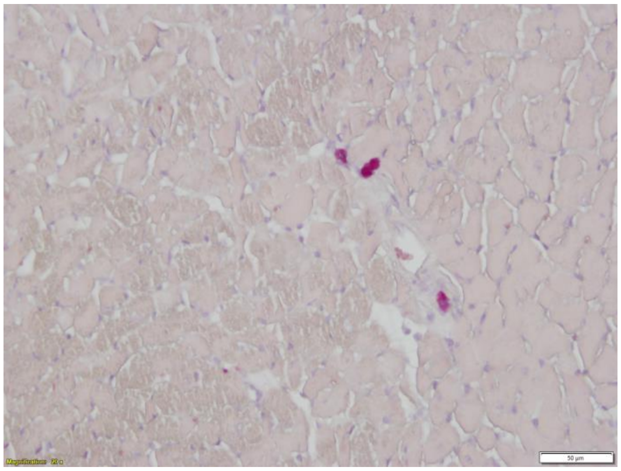

C5aR$^{-/-}$ TAC

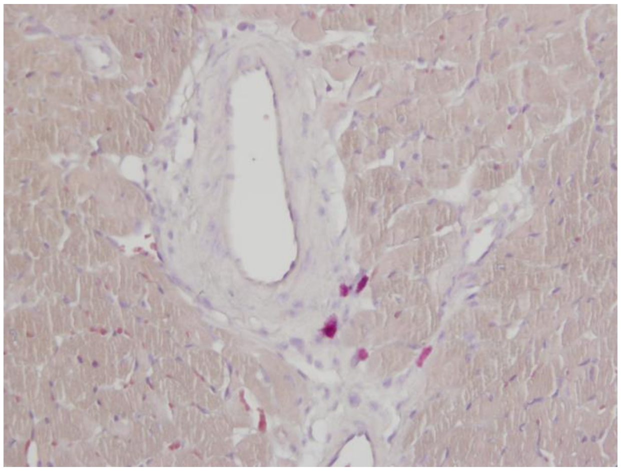

Supplement: Supplementary file 1 — Supplementary material [file 41598_2017_16957_MOESM1_ESM.pdf]
